# DNA and RNA Quadruplex-Binding Proteins

**DOI:** 10.3390/ijms151017493

**Published:** 2014-09-29

**Authors:** Václav Brázda, Lucia Hároníková, Jack C. C. Liao, Miroslav Fojta

**Affiliations:** 1 Institute of Biophysics Academy of Sciences of the Czech Republic, v.v.i., 61265 Brno, Czech Republic; E-Mails: luh@ibp.cz (L.H.); jack.liao@uq.net.au (J.C.C.L.); fojta@ibp.cz (M.F.); 2 School of Medicine, University of Queensland, Brisbane 4006, Australia; 3 Central European Institute of Technology, Masaryk University, 62500 Brno, Czech Republic

**Keywords:** DNA quadruplex, RNA quadruplex, telomere, protein–DNA binding, regulation

## Abstract

Four-stranded DNA structures were structurally characterized *in vitro* by NMR, X-ray and Circular Dichroism spectroscopy in detail. Among the different types of quadruplexes (i-Motifs, minor groove quadruplexes, G-quadruplexes, *etc.*), the best described are G-quadruplexes which are featured by Hoogsteen base-paring. Sequences with the potential to form quadruplexes are widely present in genome of all organisms. They are found often in repetitive sequences such as telomeric ones, and also in promoter regions and 5' non-coding sequences. Recently, many proteins with binding affinity to G-quadruplexes have been identified. One of the initially portrayed G-rich regions, the human telomeric sequence (TTAGGG)*_n_*, is recognized by many proteins which can modulate telomerase activity. Sequences with the potential to form G-quadruplexes are often located in promoter regions of various oncogenes. The NHE III_1_ region of the c-*MYC* promoter has been shown to interact with nucleolin protein as well as other G-quadruplex-binding proteins. A number of G-rich sequences are also present in promoter region of estrogen receptor alpha. In addition to DNA quadruplexes, RNA quadruplexes, which are critical in translational regulation, have also been predicted and observed. For example, the RNA quadruplex formation in telomere-repeat-containing RNA is involved in interaction with TRF2 (telomere repeat binding factor 2) and plays key role in telomere regulation. All these fundamental examples suggest the importance of quadruplex structures in cell processes and their understanding may provide better insight into aging and disease development.

## 1. Introduction

The discovery of the B-DNA structure was one of the most important events in natural science during the last century [1]. However, knowledge of the DNA sequences and structures has led to fascinating findings of various DNA forms that differ from the canonical right-handed Watson–Crick double–helix. These unusual DNA structures play critical roles in regulation of very basic biological functions and are integral part of the complex regulatory systems of living beings. The negative supercoiling of DNA can induce sequence-dependent conformational changes that give rise to local DNA structures and alternative DNA conformations such as cruciforms, A-DNA, left-handed DNA (Z-DNA), triplexes, four-stranded DNA (quadruplexes) and others [2,3].

### 1.1. Structure and Formation of Quadruplexes

**Figure 1:**
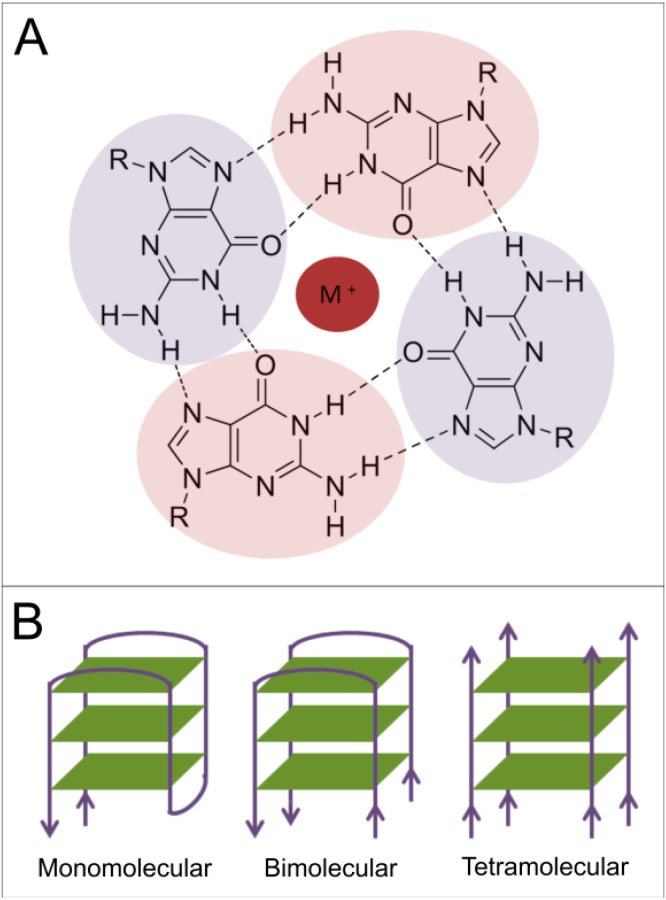
(**A**) Scheme of Hoogsteen base-paring in G-quadruplex structures. The stacked tetrads of guanines (highlighted-purple, violet) are stabilized by a metal ion (M^+^, red) in the middle of the quadruplex; and (**B**) Quadruplexes can be formed within a single nucleic acid strand, from two strands (as a dimer of hairpins) or from four separate DNA or RNA strands. Green planes represent the guanine tetrads. Grey lines represent the sugar-phosphate backbone, with the arrows showing polarity of the nucleic acid chains.

**Figure 2:**
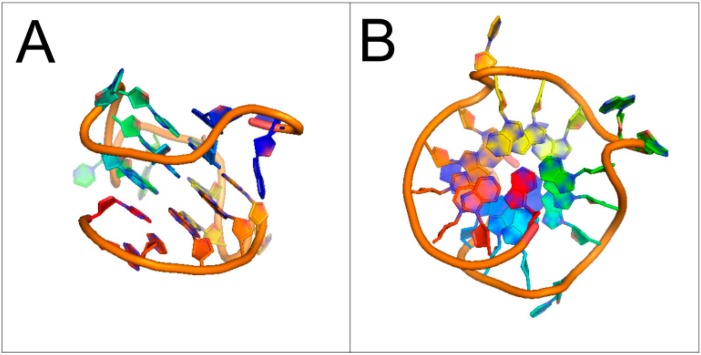
Structure of G-quadruplex in the nuclease hypersensitive element (NHE) III_1_ region of human c-*MYC* promoter (PDBid: 1XAV, [16]). (**A**) Side view; and (**B**) Bottom view. Sugar-phosphate backbone is represented by the orange ribbon, with the guanine bases forming the tetrads located in the middle.

The existence of “tetraplex nucleic acids” (quadruplexes) has been studied in great detail. Quadruplexes can originate from both DNA and RNA molecules and their formation is possible especially in sequences with high abundance of guanine (G-quadruplex) and cytosine (i-motifs). However, it was demonstrated that G-quadruplexes may contain other kinds of tetrads consisting of adenine [4], thymine [5] and cytosine [6] or mixed tetrad containing Watson-Crick base-pairing in the context of G-quadruplex [7,8]. Structure of the minor groove quadruplexes where no G-tetrads are present was also shown based on GC and AT base-pairing [9]. i-Motifs are four-stranded DNA secondary structures which are formed in cytosine-rich sequences. Stabilized by acidic conditions, they are comprised of two parallel-stranded DNA duplexes held together in an antiparallel fashion by intercalated, cytosine–cytosine base-pairing [10]. Although the potential of forming unique quadruplex structures from different nucleotides appears limitless, the most abundant and examined are the G-quadruplexes (Figure 1). According to the number of molecules involved in quadruplex formation, G-quadruplexes are classified as intramolecular and intermolecular structures [11]. The best characterized G-quadruplexes arise typically from adjacent run of guanine-rich regions. Its structure is stabilized by hydrogen bonds of guanine tetrads by Hoogsteen base-pairing (Figure 1A). The negative charge in the central channel needs to be compensated by a monovalent metal ion (usually potassium or sodium ion) [12] and the stability and folding are strongly dependent on the DNA concentration [13]. Interestingly, quadruplexes can be formed from even a single nucleic acid strand, but also from two or four separate DNA or RNA strands (Figure 1B). Quadruplexes can be created with different chain orientations (parallel or antiparallel) which are characterized by their diverse glycosidic bond angles [14]. The RNA quadruplexes are preferentially formed in parallel conformation due to anti-geometry of glycosidic bond in ribonucleosides. In contrast, the DNA G-quadruplexes can adopt both parallel and antiparallel forms and can often switch from one to another, depending on experimental and sequence conditions [15]. The great variety of G-quadruplex folding is also influenced by salt concentration, the position and length of the loop, the abundance of different nucleotides in loops and the number of tetrads [11]. The detailed structure of G-quadruplexes has been extensively studied by CD spectroscopy, NMR spectroscopy and X-ray (Figure 2).

### 1.2. Presence of Quadruplex-Forming Sequences in Genomic DNA

Self-association of guanosine has been observed since the late 19th century. However, the tetrameric arrangement of guanine bases was not determined by crystallographic methods as the G-quartet until 1962 [17]. One of the first discovered and characterized sequences forming quadruplex structure was the human telomeric sequence [18]. The presence of G-rich sequences in promoters of oncogenes was subsequently described with the structure of such sequence solved *in vitro*. Nowadays, the power of sequencing data allows analysis of genomic sequence potential to form quadruplexes. Currently, there are several tools for quadruplex prediction [19,20,21]. These software programs are capable of generating information on the composition and distribution of putative quadruplex-forming G-rich motifs in nucleotide sequences. The quadparser algorithm is able to predict quadruplex formation based on the sequence, with consideration of strand stoichiometry, the number of stacked tetrads in the quadruplex core, the presence of base substitutions or deletions, and the length and composition of loops. In the human genome, approximately 376,000 putative quadruplex sequences have been predicted [22]. Besides the telomeric and other G-rich repeats (see below), these putative quadruplexes are frequently located in promoter regions of oncogenes, suggesting their function in controlling of gene expression at the transcriptional level [19]. Moreover, presence of putative quadruplex sequences in exon region suggests a role of RNA quadruplexes in translational regulation. Expectably, quadruplex-forming sequences are not only present in the human genome, but also rich in the genomes of prokaryotes and other eukaryotes, with potential significance in cell regulation [23,24].

#### Tools for Detection of Quadruplexes

The structures adopted by many putative quadruplex sequences from the genomic DNA have been characterized using NMR, X-ray and CD spectroscopy. These methods confirmed the ability of oligonucleotide strands folding into quadruplex structures under *in vitro* conditions. The existence of these structures has also been intensively studied *in vivo* using two main approaches. First is the use small quadruplex-binding ligands which can bind to the G-quartet or to the groove and loop region [25]. Due to the great variety of quadruplex structures, very selective binding ligands can be theoretically prepared. One of the studied molecules TMPyP4 (a cationic porphyrin analogue) was described as a quadruplex-binding ligand capable of regulating telomerase activity [26] and down-regulating the expression of c-*MYC*, thus demonstrating the presence of quadruplex structure in c-*MYC* promoter region [27]. Structural studies of this molecule subsequently revealed a low affinity binding for quadruplex DNA in comparison with other DNA structures [28]. Another example of small quadruplex-binding ligand is the fluorescent biomarker BMVC (3,6-bis(1-methyl-4-vinylpyridinium) carbazole diiodide) which binds selectively to quadruplex DNA over duplex DNA. The fluorescent emission maximum for this molecule is at 575 nm for quadruplex and at 545 nm for double-stranded DNA. Using fluorescent microscopy, the fluorescence of BMVC was localized near telomeric regions suggesting the presence of quadruplex structure in human telomeres [29]. Specific structure of G-quadruplexes has inspired the development of a number of specific probes which can be used for sensitive quadruplex detection [30,31], as well as to monitor the duplex-to-quadruplex transition [32]. Some of these probes have the potential to be used as therapeutic compounds [33]. Applications of G-quadruplex selective probes have recently been reviewed by He *et al.* [34].

The second approach to localize and validate quadruplex structures *in vivo* is to use antibodies. Very few quadruplex-specific antibodies have been prepared and described [35,36,37], with none of them being commercially available. The first characterized anti-quadruplex antibody hf2 recognizes quadruplex DNA with at least 100-fold higher affinity than to double-stranded DNA [35]. BG4 is another anti-G-quadruplex antibody isolated and characterized using ELISA (Enzyme-linked immunosorbent assay). It recognizes specifically quadruplex DNA not only in vitro, but it was also used for determination and localization of the G-quadruplexes *in vivo* [38]. ELISA approach showed that BG4 antibody binds with nanomolar affinity to both DNA G-quadruplexes, as well as several RNA G-quadruplexes [39]. Using fluorescent microscopy, it was demonstrated that G-quadruplexes are located mainly in the nuclear foci, with less intensive punctate staining distributed throughout the cytoplasm at longer exposures, suggesting RNA G-quadruplex recognition in the cells [39]. Notably, BG4 antibody localizes to telomere ends and is observed to associate strongly with DNA during replication [38].

## 2. Proteins Involved in Interactions with Quadruplex DNA

Bioinformatics has shown a tremendous potential of quadruplex formation in both prokaryotic and eukaryotic genome and transcriptome [24]. Moreover, these putative structures are non-randomly located with clusters in the promoter and telomeric regions. A large number of proteins which bind specifically to other non-B DNA structures (e.g., cruciforms) has also been characterized [40]. It is therefore not surprising that quadruplexes are specifically recognized by different quadruplex-binding proteins (Table 1). Because the quadruplex structure in the DNA must be unfolded during replication to allow the DNA polymerase to read the template strand sequence, any error in quadruplex recognition could lead to extensive replication errors, DNA damage and reorganization of chromosomes. Thus, we suspect that the number of known proteins with quadruplex-binding specificity and/or quadruplex-helicase activity will be rapidly increased in the future.

**Table 1:** Proteins involved in quadruplex binding and resolving.

Localization/Function	Gene	Protein	Reference
**Telomere Region**		BRCA1	[41]
		hnRNP A1	[42]
		hnRNP D	[43]
		POT1	[44,45,46,47]
		RPA	[47,48]
		TEBPs	[49,50]
		TLS/FUS	[51]
		Topo I	[52]
		TRF2	[53]
		UP1	[54]
**Promoter Regions**	*BCL-2*	PARP1	[55]
	*c-MYC*	CNBP	[56]
	*c-MYC*	nucleolin	[57]
	*c-MYC*	nucleophosmin	[58]
	*Insulin*	IGF-2, insulin	[59]
	*KRAS*	hnRNP A1	[60]
	*KRAS*	MAZ	[61,62]
	*KRAS*	PARP1	[55,62]
	*MYB*	PARP1	[55]
	*KIT*	PARP1	[55]
	*VEGF*	PARP1	[55]
		Mutant p53 protein	[63]
		MutSα	[64]
		Topo I	[52]
**RNA Quadruplexes**		FMR2	[65,66]
		hnRNP A1 mutant	[67]
		hnRNP A2	[67,68]
		nucleolin	[69]
		RHAU	[70]
		Ribosomal proteins	Reviewed in [69]
		SRSF 1 and 9	[69]
		TLS	[51]
		TRF2	[53]
**Quadruplex-Resolving Helicases**		BLM	[71,72]
		Dna2	[73]
		FANCJ	[74]
		G4R1/RHAU	[75,76]
		Sgs1	[77]
		WRN	[78,79]

BRCA1, breast cancer type 1 susceptibility protein; hnRNP, heterogeneous nuclear ribonucleoprotein; POT1, protection of telomeres 1; RPA, replication protein A; TEBP, Telomere End Binding Protein; TLS/FUS, translocated in liposarcoma/fused in sarcoma; Topo I, Topoisomerase I; TRF2, telomere repeat binding factor 2; UP1, unwinding protein 1; PARP-1, Poly [ADP-ribose] polymerase 1; CNBP, cellular nucleic-acid-binding protein; IGF-2, Insulin-like growth factor 2; MAZ, myc-associated zinc-finger; FMR2, fragile X mental retardation 2; RHAU, the RNA helicase associated with AU-rich element; SRSF, serin/arginine-rich splicing factor; BLM, Bloom syndrome protein; Dna2, DNA replication helicase/nuclease 2; G4R1, G4 Resolvase 1; FANCJ, Fanconi anemia complementation group J; Sgs1, small growth suppressor 1; WRN, Werner syndrome ATP-dependent helicase.

### 2.1. Telomeric Quadruplex-Binding Proteins

One of first discovered and characterized sequences forming quadruplex structure was the human telomeric sequence [80]. Telomeres play crucial roles in genome integrity and stability, mainly via counterbalancing the replicative shortening of the chromosomal ends. The enzyme telomerase maintains G-rich strand 3'-overhang of about 20 (yeast)–200 (human) nucleotides which would be shortened after each replication cycle by 50–100 nucleotides. [81]. The chromosomal ends are protected by a capping process, where specific proteins bind to telomeric DNA to prevent not only nuclease degradation, but also recognition of the chromosomal ends as double-strand breaks by the DNA repair machinery [82]. Other processes such as T-loop formation [83] and G-quadruplex formation [84] have also been described to play roles in protecting the 3'-overhangs.

Telomeric DNA of eukaryotic organism consists of repetitive sequences with the potential to form quadruplex structures. Some organisms have remarkably G-rich sequences conferring the formed G-quadruplexes a high stability, especially in sequences with three or more consecutives guanines. The most stable G-quadruplex appears to be formed by the telomeric sequence of *Tetrahymena*, harbouring 22 nucleotides with TTGGGG repeat unit. On the contrary, yeast telomeric DNA containing two consecutive guanines in a repeat unit does not prefer the quadruplex formation [85]. Human telomeric DNA consists of tandem repeats of the sequence d(TTAGGG). In physiologically relevant conditions, the parallel and also the antiparallel arrangements are possible due to different DNA concentration in solution [12]. The presence of quadruplex structure in the telomere region *in vivo* was confirmed by several experiments using quadruplex-specific antibodies [35,38] and quadruplex-binding ligands [29,86].

#### 2.1.1. Proteins of Shelterin Complex

Shelterin is a complex composed of six proteins and is an integral part of vertebrate telomeres. Shelterin proteins are essential for proper telomere function in the maintenance of genomic stability including impact to telomerase activity. Moreover, it is involved in protecting human telomeric DNA from being recognized as a chromosomal break. The shelterin proteins include: POT1 (protection of telomeres 1), TRF1 and TRF2 (telomere repeat binding factor 1 and 2), TPP1, TIN2 (TRF1 interacting protein 2) and RAP1 (repressor activation protein 1). TRF1 and TRF2 contain two main domains: *C*-terminal DNA binding domain and a homodimerization domain. Their protein structures determine their preference for double-stranded DNA [87,88]. POT1 is a single-stranded DNA-binding protein that binds to 3'-overhang of telomeric repeats, but not to the double-stranded telomeric sequence or the C-rich sequence [89]. The main role of human POT1 is to prevent activation of ATR kinase signalling, thereby protecting chromosomal ends from being seen as DNA damage [90]. TPP1 is a heterodimeric partner of POT1, capable of enhancing the POT1-DNA interaction [91] and regulating the access of telomerase to 3'-overhang [92,93].

#### 2.1.2. Telomere End Binding Proteins

**Figure 3:**
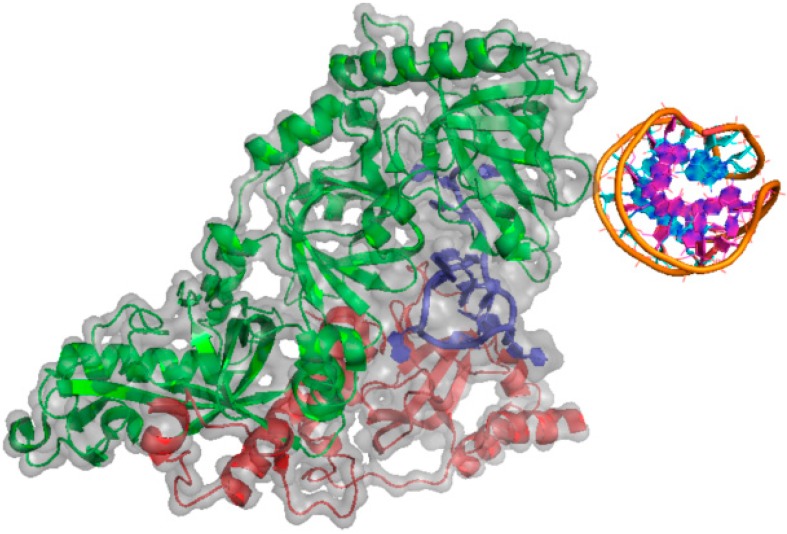
Structure of the DNA G-quadruplex of an *Oxytricha nova* telomeric protein-DNA complex (PDBid: 1JB7) [50]. Sugar-phosphate backbone and nucleobases in the DNA quadruplex structure is depicted by the orange ribbon and purple/cyan cartoons, respectively. The α- and β-subunits of the G-quadruplex-binding protein are represented by the green and red cartoons, respectively. A single-stranded DNA is represented by the blue cartoon. Grey color highlights the electron cloud of the protein-DNA complex.

POT1-TPP1 interaction is analogous to the α-β heterodimer of the telomere end binding protein (TEBP) described in *O. nova* [91,94]. The latter protein complex (PDBid 1JB7, Figure 3) binds to 3'-overhang in the form of a quadruplex [50]. In 2012, Hwang *et al.* [45] depicted the role of G-quadruplex structure in relation with POT1 and in complex with TPP1. Using single molecule fluorescence assay, they reported a very interesting mechanism of interaction with the quadruplex structure. Protein POT1 approaches G-rich sequence in 3'–5' direction and initiates the G-quadruplex unfolding in two steps. Subsequent complex formation with TPP1 results in unfolding and refolding of the G-quadruplex. The sliding activity of POT1–TPP1 complex allows enhancement of telomerase processivity.

The unfolding activity of G-quadruplex structure has been observed also for replication protein A (RPA), which is involved in replication, repair and recombination [48,95]. The mechanism of unfolding involves a two-step process [48], with approaching of strand in 5'–3' direction, opposite to that of POT1-TPP1 protein complex [47]. Although both RPA and POT1-TPP1 are able to bind to telomere overhang, RPA is more abundant in the cell. The mechanism of telomere overhang protection by POT1-TPP1 *versus* RPA was studied by Ray *et al.* [46] in 2014 using single-molecule fluorescence resonance energy transfer (smFRET). It was shown that POT1 unfolds G-quadruplexes in antiparallel conformation while the parallel orientation remains stable. RPA protein on the other hand, unfolds both conformations of G-quadruplexes. Access of RPA protein to telomeric DNA is blocked by both the presence of POT1-TPP1 and G-quadruplexes. Thus, the presence of quadruplex and its binding by POT1 enhances protection against RPA interaction.

Recognition of G-quadruplexes by specific proteins provides functional evidence of these structures *in vivo*. As previously reported, the heterogeneous nuclear ribonucleoprotein A1 (hnRNP A1) and its proteolytic derivative, unwinding protein 1 (UP1), bind to and destabilize G-quadruplex structures formed by the human telomeric repeat d(TTAGGG)*_n_*. This interaction has a binding affinity that is 200 times stronger than the binding of UP1 to a single-stranded DNA with a comparable but non-quadruplex-forming sequence [54].

#### 2.1.3. BRCA1 (Breast Cancer Type 1 Susceptibility Protein)

It has been demonstrated that BRCA1 protein binds to structures that are stabilized by superhelical coiling such as cruciforms [96]. Importantly, BRCA1 protein interacts directly with the human telomeres and regulates telomerase activity and the length of telomeric 3'-overhang [41]. This was established by telomeric ChIP (chromatin imunoprecipitation) assay and confocal microscopy, showing colocalization of BRCA1 protein with telomeric DNA in cultured cells [97]. Recently, it was observed that BRCA1 mutation carriers have longer telomeres than their non-mutation carriers [98]. Moreover, BRCA1 is repeatedly absent or significantly decreased in sporadic breast cancer [99]. Given BRCA1 protein’s newly identified role in telomere regulation [41], its preferential binding to quadruplex DNA may indicate an important role in processes which are associated with quadruplex formation in the genome.

Processes that take place at the telomeres are very complex and their regulations likely involve cooperation of multiple proteins. Although the exact mechanism of telomere regulation remains unclear, the presence of quadruplex structure and its particular conformation at the telomeres has been shown to play a critical role.

### 2.2. Proteins Involved in Transcription and Binding G-Quadruplexes in Promoter Regions

Using *in silico* analyses of the genome it was revealed that huge amount of potential G-quadruplex-forming motifs (G≥3N1-7) ≥4 are clustered in promoter regions [22]. The quadruplex formation was present in promoter regions of many important genes associated with oncogenesis, including one of the most commonly malfunctioned genes in human cancers: c-*MYC* oncogene [100]. It was also demonstrated that G-quadruplex motif from the human estrogen receptor α gene (ESR1) can modulate the efficiency of translation [101]. These findings indicate a role of quadruplex structure in regulation of transcription. It is therefore not surprising that among quadruplex-binding proteins (Table 1), many of them are involved in transcriptional regulation (e.g., poly [ADP-ribose] polymerase 1 (PARP-1) and mutant p53 protein), as well as in chromatin remodeling and DNA repair (e.g., nucleolin, nucleophosmin, BRCA1 tumor suppressor, CNBP (cellular nucleic acid-binding protein), MAZ (myc-associated zinc-finger) protein and hnRNP A1).

#### 2.2.1. PARP-1 (Poly [ADP-ribose] polymerase 1)

PARP-1 is an abundant nuclear zinc-finger protein present in approximately one in every 50 nucleosomes. It has a high affinity for damaged DNA and becomes catalytically active upon binding to DNA breaks [102]. PARP-1 activity is also linked to coordination of chromatin structure and gene expression [103]. It was reported that PARP-1 can bind to DNA hairpins and promoter region in superhelical DNA and promote formation of cruciform structure [104]. PARP-1 recognizes distortions in the DNA backbone allowing it to interact with three- and four-way junctions [105]. Soldatenkov *et al.* demonstrated that PARP-1 binds to intramolecular DNA quadruplexes *in vitro* with high affinity and with a stoichiometry of two proteins for one quadruplex [55]. Using an enzymatic assay, it was shown that PARP-1 gets catalytically activated upon binding to G-quadruplexes localized at the c-kit promoter [55]. Pull-down and chromatin immunoprecipitation assays revealed that this quadruplex-forming element is bound by MAZ and PARP-1 proteins [62]. It was also found that using plasmid DNA targets, binding of PARP-1 to DNA can induce changes in DNA topology [104]. Furthermore, a critical quadruplex-forming element of murine KRAS oncogene interacts with MAZ and PARP-1 proteins via conformational changes from duplex to quadruplex DNA. Importantly, both MAZ and PARP-1 are activators of the KRAS promoter and recognize parallel quadruplex conformation adopted by the quadruplex-forming element, which likely is a key in recruiting these proteins to the promoter [55,61].

#### 2.2.2. Mutant p53 Protein

P53 is one of the most extensively studied tumor suppressor genes. More than 50% of all human tumors contain p53 mutations and inactivation of this gene plays a critical role in the induction of malignant transformation [106]. While p53, as a transcription factor, binds sequence-specifically to DNA target sites, its preference for superhelical DNA and cruciform structures has also been described [107,108]. Furthermore, p53 binding to mismatched duplexes, cruciform structures [109,110,111], bent DNA [112], structurally flexible chromatin DNA [113], hemicatenated DNA [114], DNA bulges, and three- and four-way junctions [115] has been demonstrated. Rapid disease progression of the oncology patients carrying p53 mutation leads to the theory that the mutation not only brings about loss of the protective role of the wild-type p53 protein, but also acquires a new function—also known as p53 mutant “gain of function”. The molecular mechanism of this new oncogenic role of mutant p53 remains unclear however. Recently, it was reported that mutant p53 protein can preferentially binds to C-G rich DNA sequences and stabilize G-quadruplex structure *in vitro* [63]. These results implicate that DNA topology could play a role in mutant p53 protein’s “gain of function” character and that quadruplexes are its preferential targets.

#### 2.2.3. Nucleolin and Nucleophosmin

Nucleolin is a nucleolar phosphoprotein expressed highly in proliferating cells with multiple roles in ribosome biogenesis [116], chromatin remodeling [117], transcription [118], G-quadruplex binding [119], and apoptosis [120]. Interestingly, nucleolin-hnRNP D heterodimer has been reported to bind to G-quadruplex structures [119,121]. It was shown that overexpression of nucleolin can significantly inhibit c-*MYC* promoter-driven transcription as measured by luciferase activity in MCF10A cells. Nucleolin binds *in vitro* to the c-*MYC* G-quadruplex structure with high affinity and selectivity when compared with other known quadruplex structures. In addition, nucleolin facilitates the formation of the c-*MYC* G-quadruplex structure and increases its stability. Importantly, it was also revealed that nucleolin binds to the c-*MYC* promoter *in vivo* [62].

Nucleophosmin is another multifunctional protein with implications in the pathogenesis of several human malignancies. This protein exerts its function through interaction with different protein partners including p53, p14arf, *etc.* It specifically recognizes G-quadruplexes through its intrinsically unfolded *C*-terminal region [122] which contributes largely to the binding of c-*MYC* G-quadruplex motif [58].

### 2.3. RNA Guanine Quadruplex-Binding Proteins

The formation of G-quadruplexes in G-rich regions of RNA can be expected to occur even more easily than in DNA since RNA is naturally single-stranded. Recently, it was shown that G-quadruplexes in RNA play a role in various cellular functions including termination of transcription, telomerase activity, alternative splicing and modulation of translation [123,124]. Using the pull-down assay, a set of proteins involved in interaction with RNA G-quadruplexes was identified and characterized, including hnRNP, ribosomal proteins, splicing factors and others [69]. Due to structural similarities between DNA and RNA G-quadruplexes, their binding proteins overlap significantly. Among the G-quadruplex-binding proteins are nucleolin, hnRNP, serin/arginine-rich splicing factor (SRSF) 1 and 9, splicing factor U2AF, ribosomal proteins [69], as well as TLS [51], TRF2 [53], FRM2 [65,66], and the RNA helicase associated with AU-rich element (RHAU) proteins [70]. Telomeres have been considered to be transcriptionally silent, but recently it was demonstrated that telomeres are transcribed into telomeric repeat-containing RNA (TERRA) [125]. Interestingly, it was also described that human telomeric RNA and telomeric DNA sequences can form hybrid parallel G-quadruplex structure [126] and could be bound by TRF2 and TLS/FUS protein complexes [53].

#### 2.3.1. hnRNPs (Heterogeneous Nuclear Ribonucleoproteins)

The hnRNP proteins are believed to play critical roles in packaging, transport and splicing of the pre-mRNA into functional complexes. The binding partners of the RNA G-quadruplexes include several hnRNPs [69]. The hnRNP A1 protein has been demonstrated to accompany the mRNA into the cytoplasm and is also present during translation. Furthermore, it was shown that hnRNP A2 destabilized the RNA tetraplex structure of the repeat sequence (CGG)*_n_* of the fragile X mental retardation 1 (FMR1) gene [67]. Interestingly, the levels of FMR1 mRNA are 5- to 10-fold higher in fragile X syndrome pre-mutation carriers with >55–200 repeats than in normal subjects. It was revealed that a RNA tract with a largely RNase T1-resistant intramolecular secondary structure was formed in the presence of K^+^ ions. Expression of the quadruplex (CGG)*_n_* disrupting proteins hnRNP A2 or its related protein CBF-A in HEK293 cells significantly elevated the efficacy of (CGG)99 mRNA translation. These results suggest that secondary structures of (CGG)*_n_* in mRNA obstruct its translation and that quadruplex-disrupting proteins alleviate the translational block [68].

#### 2.3.2. The AFF Family

The AFF (AF4 (ALL1 (acute lymphoblastic leukemias)-fused gene from chromosome 4)/FMR2 (fragile X mental retardation 2)) family of genes includes four members: AFF1/AF4, AFF2/FMR2, AFF3/LAF4 and AFF4/AF5q31. While AFF2/FMR2 is silenced in Fragile XE syndrome (FRAXE) associated with mental retardation, the other three members have been reported to form fusion genes as a consequence of chromosome translocations with the myeloid/lymphoid or mixed lineage leukemia (*MLL*) gene in acute lymphoblastic leukemias (ALLs). AFF proteins are localized in the nucleus. In particular, AFF2/FMR2 localizes to nuclear speckles which are subnuclear structures acting as storage/modification sites for pre-mRNA splicing factors. In addition, AFF2/FMR2 modulates alternative splicing via the interaction with the G-quadruplex RNA-forming structure. Other members of the AFF family are also capable of binding RNA with a high apparent affinity for G-quadruplex structures. Interestingly, similar to AFF2/FMR2, AFF3/LAF4 and AFF4/AF5q31 modulate *in vivo* the splicing efficiency of a mini-gene containing a G-quadruplex structure in one alternatively spliced exon [65].

#### 2.3.3. Ribosomal Proteins

It has been reported that RNA quadruplexes play a role in translational modulation. For example, the 5'-UTR (5' untranslated region) G-quadruplexes represent a class of translational repressors broadly distributed in the cell. Single-nucleotide polymorphisms in the 5'-UTR were also demonstrated to be important in the formation of G-quadruplexes and in modulating translational repression. Recently, it was revealed that RNA quadruplexes interact with several ribosomal proteins [69]. Some of these ribosomal proteins are involved in 43S pre-initiation complex that move along the mRNA in search of the start codon. It is likely that G-quadruplexes at 5'-UTR inhibit the recognition of the start codon due to steric hindrance and/or direct binding of proteins to quadruplex structure.

## 3. Role of Quadruplexes in Aging and Diseases

**Figure 4:**
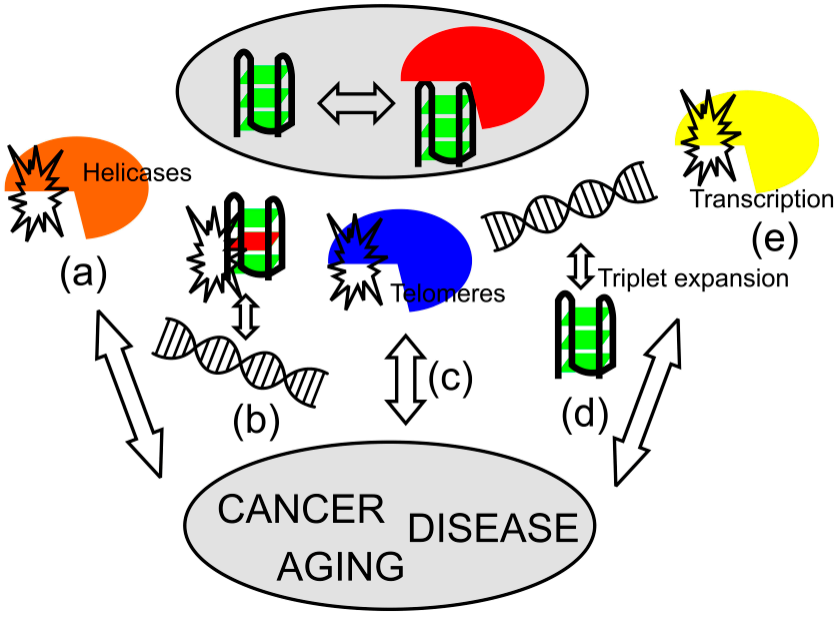
Scheme illustrating suggested roles of quadruplexes. Quadruplex formation and recognition have various functions in biological processes and their dysregulation may be associated with human disorders, as seen from mutations in quadruplex-recognition proteins, quadruplex-resolving helicases (**a**) and quadruplex-forming sequences (**b**); as well as changes in the binding affinity and stability of telomere complexes (**c**); and generation of new quadruplex-forming motifs via triplet expansions (**d**); and transcriptional alteration (**e**). Arrows show connection of changes associated with quadruplex formation and recognition and influence of these changes to aging and diseases progression.

The strong implication of putative quadruplex structures in telomere sequences and oncogene promoter regions, as well as the formation of non-B DNA structure in triplet expansion-associated diseases all hinted the importance of quadruplex structures in processes associated with aging and diseases development, as illustrated in Figure 4.

### 3.1. Aging Processes

Aging processes are closely related to telomere shortening, decreased DNA reparation ability, decreased activity of regeneration processes, and increased risk of disease development. In this view, the telomere capping process by quadruplex-binding proteins is of critical importance in telomere stability [127]. On one side, it is clear that telomere shortening is a process leading to aging, but on the other side, cancer cells have often regained telomere capping capability or have gained an unlimited proliferation potential through the continued expression of telomerase [128]. Some cancer cells have short telomeres that remain stable over time. These cells may have defective capping capacity or may harbor somatic mutations in telomere capping components, thereby driving further genomic instability and tumor evolution. Fine-tuning of telomeres is therefore a crucial step in aging and cancer development. Interestingly, statistical analyses showed association of age-related cancer onset in TP53 mutation carriers with polymorphisms in predicted G-quadruplex structures [129]. This result implicates that even polymorphisms and mutations in sequence which influence the presence and formation of non-B DNA structures can be important in the development of cancer and aging. The G-quadruplex structural motif of DNA has emerged as a novel and exciting target for anticancer drug discovery. Small molecules that selectively target and stabilize the G-quadruplex structure may serve as potential therapeutic agents and have garnered significant interest in recent years. For example, the anticancer agent actinomycin D binds to and induces changes in both the structure and stability of the G-quadruplex DNA [106].

### 3.2. Disorders Associated with G-Quadruplex Helicases

G-quadruplex helicases normally act to resolve quadruplexes before replication to maintain genomic stability. Malfunctions of G-quadruplex helicases are thus associated with human disorders. For example, ChlR1 helicase is genetically linked to Warsaw Breakage Syndrome which is a chromosomal instability disorder characterized by cohesion defects and sensitivity to DNA cross-linking agents [16]. FANCJ (Fanconi anemia complementation group J) family of DNA helicases including mouse RTEL(regulator of telomere length) and nematode DOG-1 (discovered on GIST-1) have been implicated in telomere maintenance [130]. FANCJ-depleted human cells are sensitive to quadruplexes and have elevated DNA damage and apoptosis upon exposure to the G4-interactive compound telomestatin [74]. Moreover, FANCJ-deficient cells accumulate deletions at genomic sequences with a G4 DNA signature [131], suggesting that FANCJ prevents replication-associated DNA damage by removing G4 structures. It was reported that WRN (Werner syndrome ATP-dependent helicase) and BLM (Bloom syndrome protein) helicases are also capable of efficiently unwinding G-quadruplexes *in vitro* [132]. This step of quadruplex unwinding is essential during DNA replication, as mutations of WNR and BLM helicases are associated with Werner syndrome and Bloom’s syndrome, respectively. Furthermore, WNR not only is important for achieving telomeric stability, but also interacts with shelterin proteins. Taken together, these results underscore the value of dynamic regulation of quadruplexes in the cellular context. Moreover, the growing number of helicases implicated in the maintenance of genomic stability suggests multiple layers of complexity in terms of their involvement.

### 3.3. Triplet Repeat Disorders

It has been proposed that quadruplex formation in triplet repeats may be responsible for disturbing the DNA metabolism with subsequent genetic instabilities associated with repeat expansion [133]. Expansion of a (CGG)*_n_* sequence in the 5'-UTR of the FMR1 gene to >200–2000 repeats abolishes its transcription and initiates fragile X syndrome. It was noted that the (CGG)*_n_* motif which is typical for fragile X syndrome is capable of forming several DNA secondary structures, including quadruplexes. The quadruplex formation is supported by the finding that the CGG repeats blocked DNA synthesis *in vitro* specifically in the presence of K^+^ ions [25]. In contrary, it was also determined that G-rich fragile X DNA repeat does not form a quadruplex structure under physiological conditions [134]. FRAXE is an intellectual disability syndrome associated with silencing of the *FMR2*, which lies close the *FMR1* gene on the X chromosome. The expansion and hypermethylation of the CCG repeats in the 5'-UTR of *FMR2* has been noted to cause non-specific forms of mental disability. The cellular function of FMR2 protein is presently unknown. Using analogy with its homologous protein AF4, FMR2 is believed to play a role in transcriptional regulation, although robust evidence supporting this hypothesis is lacking. FMR2 has been shown to co-localize with the serine/arginine-rich splicing factor 2 in nuclear speckles, the nuclear regions where splicing factors are concentrated, assembled and modified [66]. FMR2 can also localize to the nucleolus when splicing is blocked. Moreover, FMR2 is able to specifically bind the G-quartet-forming RNA structure with high affinity [66]. These findings suggest that FMR2 is an RNA-binding protein, which could be involved in alternative splicing regulation through interaction with the RNA quadruplex structure.

### 3.4. Quadruplexes and Oncogenesis

It has been demonstrated that G-rich DNA can form G-quadruplex structures in the promoter region of human genes and 5' non-coding regions [20]. Recent findings show that stabilization of G-quadruplex structures can silence DNA transcription, which strongly suggests that G-quadruplex structures and their binding proteins are directly involved in the regulation of gene expression. For instance, formation of G-quadruplex in c-*MYC* oncogene has been studied with implications in cancer therapy [100]. Furthermore, G-quadruplex in c-*MYC* oncogene is a target for several G-quadruplex-binding proteins [135]. Another example illustrating the link between quadruplexes and oncogenesis is that several insulin-linked polymorphic region (ILPR) variants form G-quadruplex DNA structures *in vitro* that exhibit binding affinity to insulin and IGF-2. It has been suggested that the ILPR may form G-quadruplexes *in vivo* as well, thus raising the possibility that insulin and IGF-2 may bind to these structures in the chromatin of live cells [59]. Last but not least, binding and transcriptional activation of KRAS oncogene by MAZ and PARP-1 proteins provide the basis for rationale design of anticancer drugs. For example, the G-rich G4-aptamer which is specific for MAZ and PARP-1 has been reported to down-regulate the expression of oncogenic KRAS in cancer cells [136].

### 3.5. Presence of Quadruplexes in Viral Genomes

Interestingly, G-quadruplexes have been found as important structural elements in viral genomes. It was demonstrated that G-quadruplex could be important for internal ribosomal entry site (IRES) initiation of translation [137]. IRES elements were first found in viruses [138,139,140], but later identified also in mRNA of proto-oncogenes, growth factors, transcription and translation factors, *etc.* [137,139,141]. G-quadruplex structures were recognized, for example, in the Epstein-Barr virus-encoded nuclear antigen 1 (EBNA1) mRNA [142], as well as in all the known human papillomaviruses [143]. Moreover, G-quadruplex was also reported recently in the U3 region of the HIV-1 virus [144]. Taken together, it is likely that quadruplexes may play important roles in the control of viral replication, transcription, translation and/or recombination.

## 4. Conclusions

The large number of potential quadruplex structures in all genomes pointed to their importance in cell regulation. Epigenetic modifications and alternative DNA structures appear to provide a higher level of information which may determine and fine-tune complex biological processes at the molecular level. Local DNA structures including cruciforms, triplexes and quadruplexes are often formed in the domains of negatively supercoiled DNA and they could be stabilized and regulated by protein interaction. Since these structures could also be the source of genomic instability, they have to be tightly regulated especially during DNA replication. Telomeric quadruplexes can contribute to the protection of the chromosomal ends. G-quadruplexes in promoter regions can also influence transcription efficiently. Association of quadruplexes with oncogenic and tumor suppressor proteins suggests that quadruplexes may play roles in cancer development and are possible targets for gene therapy. Quadruplex-binding proteins can be divided into several categories. In addition to a well characterized group of proteins which bind specifically to telomeric DNA, we have further classified quadruplex-binding proteins into those which bind to DNA quadruplexes, and those which associate with RNA quadruplexes (see Table 1). Using a new computational tool for examination of conserved G-quadruplex motifs, a great deal of G-quadruplexes conserved across species was identified [145]. Stability of the quadruplexes in evolution suggests the significance of these structures. A deeper understanding of the processes related to their formation, function and recognition will be an important piece of the puzzle in providing better insight into the regulation of living organisms.
